# ^26^Al/^10^Be Burial Dating of Xujiayao-Houjiayao Site in Nihewan Basin, Northern China

**DOI:** 10.1371/journal.pone.0118315

**Published:** 2015-02-23

**Authors:** Hua Tu, Guanjun Shen, Haixu Li, Fei Xie, Darryl E. Granger

**Affiliations:** 1 College of Geographical Sciences, Nanjing Normal University, Nanjing, China; 2 Hebei Provincial Institute of Cultural Relics, Shijiazhuang, China; 3 Department of Earth, Atmospheric, and Planetary Sciences, Purdue University, West Lafayette, Indiana, United States of America; 4 Department of Natural Science, Zhangjiakou University, Zhangjiakou, China; University of Oxford, UNITED KINGDOM

## Abstract

The Xujiayao-Houjiayao site in Nihewan Basin is among the most important Paleolithic sites in China for having provided a rich collection of hominin and mammalian fossils and lithic artifacts. Based on biostratigraphical correlation and exploratory results from a variety of dating methods, the site has been widely accepted as early Upper Pleistocene in time. However, more recent paleomagnetic analyses assigned a much older age of ∼500 ka (thousand years). This paper reports the application of ^26^Al/^10^Be burial dating as an independent check. Two quartz samples from a lower cultural horizon give a weighted mean age of 0.24 ± 0.05 Ma (million years, 1σ). The site is thus younger than 340 ka at 95% confidence, which is at variance with the previous paleomagnetic results. On the other hand, our result suggests an age of older than 140 ka for the site’s lower cultural deposits, which is consistent with recent post-infrared infrared stimulated luminescence (pIR-IRSL) dating at 160–220 ka.

## Introduction

**Fig 1:**
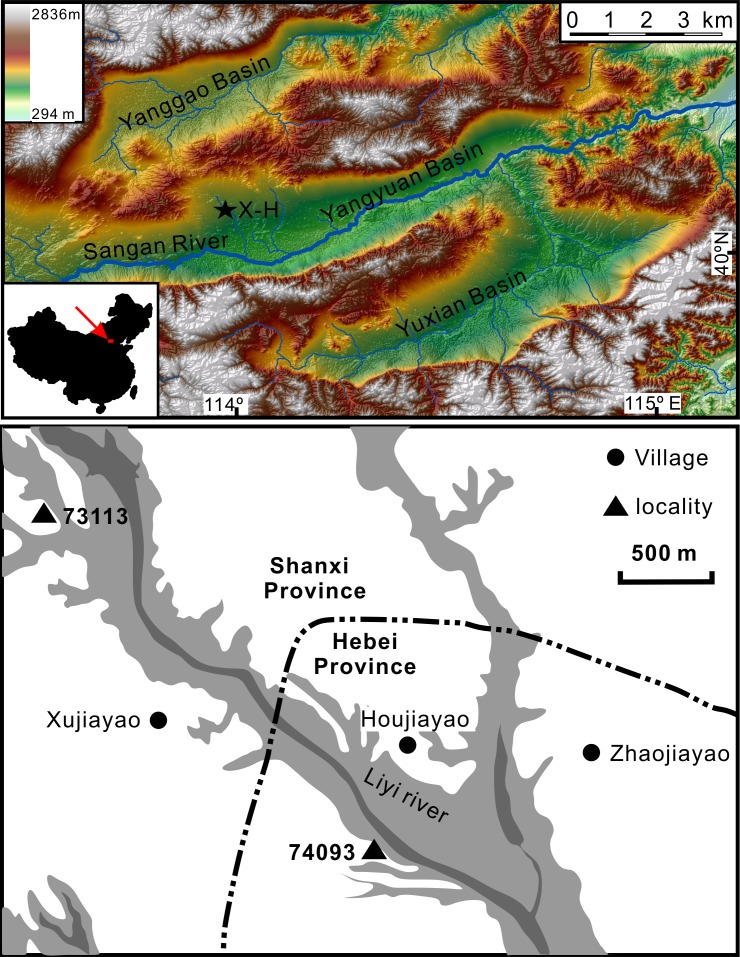
Location of the Xujiayao-Houjiayao Paleolithic site. The upper sketch shows the topographic map of Nihewan Basin and the neighboring Yanggao and Yuxian Basins. The lower one shows the positions of the Localities 73113 and 74093 of the site (modified from Wang et al. [29]).

The Paleolithic site Xujiayao-Houjiayao is located at the northwestern margin of the Nihewan Basin, northern China (Fig. 1). The site is comprised of two localities: 73113 at Xujiayao Village in Yanggao County, Shanxi Province, and 74093 at the neighboring Houjiayao Village in Yangyuan County, Hebei Province. While the first stone artifacts were discovered at the former locality, it is from the latter locality that an overwhelming majority of the archaeological materials have been recovered, which include 19 hominin fossils traditionally assigned to archaic *Homo sapiens*, more than 30,000 lithic artifacts and an abundance of mammalian fossils [1–5]. Almost all of the publications and chronological studies have dealt with the latter locality. This explains why the site was initially named Xujiayao, but later renamed Xujiayao-Houjiayao by the State Administration of Cultural Heritage of China and simply as Houjiayao by the archaeologists in Hebei Province. Here we will follow its formal nomenclature as Xujiayao-Houjiayao (hereafter X-H), but refer specifically to Locality 74093. With an exceedingly rich recovery of archaeological and fossil remains the X-H site is among the most important Paleolithic sites in China. Its precise chronological position is important for clarifying highly debated issues of Middle Pleistocene human evolution in China.

Based on faunal composition Jia and Wei [1] first broadly attributed the site to the Upper Pleistocene. Later, with the discovery several meters above the cultural deposits of a strongly deformed periglacial involution layer, believed to be formed during the last glacial, Jia et al. [2] proposed a more specific age of >100 ka. Chen et al. [6] performed alpha spectrometric ^230^Th/^234^U and ^231^Pa/^235^U dating of six fossil teeth. As the two U-series methods gave inconsistent age results, the samples were judged to have experienced open-system behavior, and the ^230^Th/^234^U ages (∼100 ka) were suggested to represent the site’s minimum age [6]. Later, based on the “nearly concordant” ^230^Th/^234^U and ^231^Pa/^235^U ages from another rhinoceros tooth, Chen et al. [7] assigned a bracket of 104–125 ka to the site. Conventional ^14^C dating was applied to sediment samples rich in organic materials, yielding minimum ages of >40 ka [8, 9]. This was confirmed by recent AMS ^14^C dating of fossil teeth (>54 ka) [10] and Infrared Stimulated Luminescence (IRSL) dating of feldspar grains (>60–69 ka) [11]. In addition, Liu et al. [12] performed a magnetostratigraphic study of the site. A short zone (∼1 m thick) with reversed geomagnetic polarity ∼3 m below the cultural layer was believed to mark the Blake excursion (117 ka). With all the geochronological information considered together, the X-H site has been widely accepted by the prehistoric community as early Upper Pleistocene in time [5, 13].

**Fig 2:**
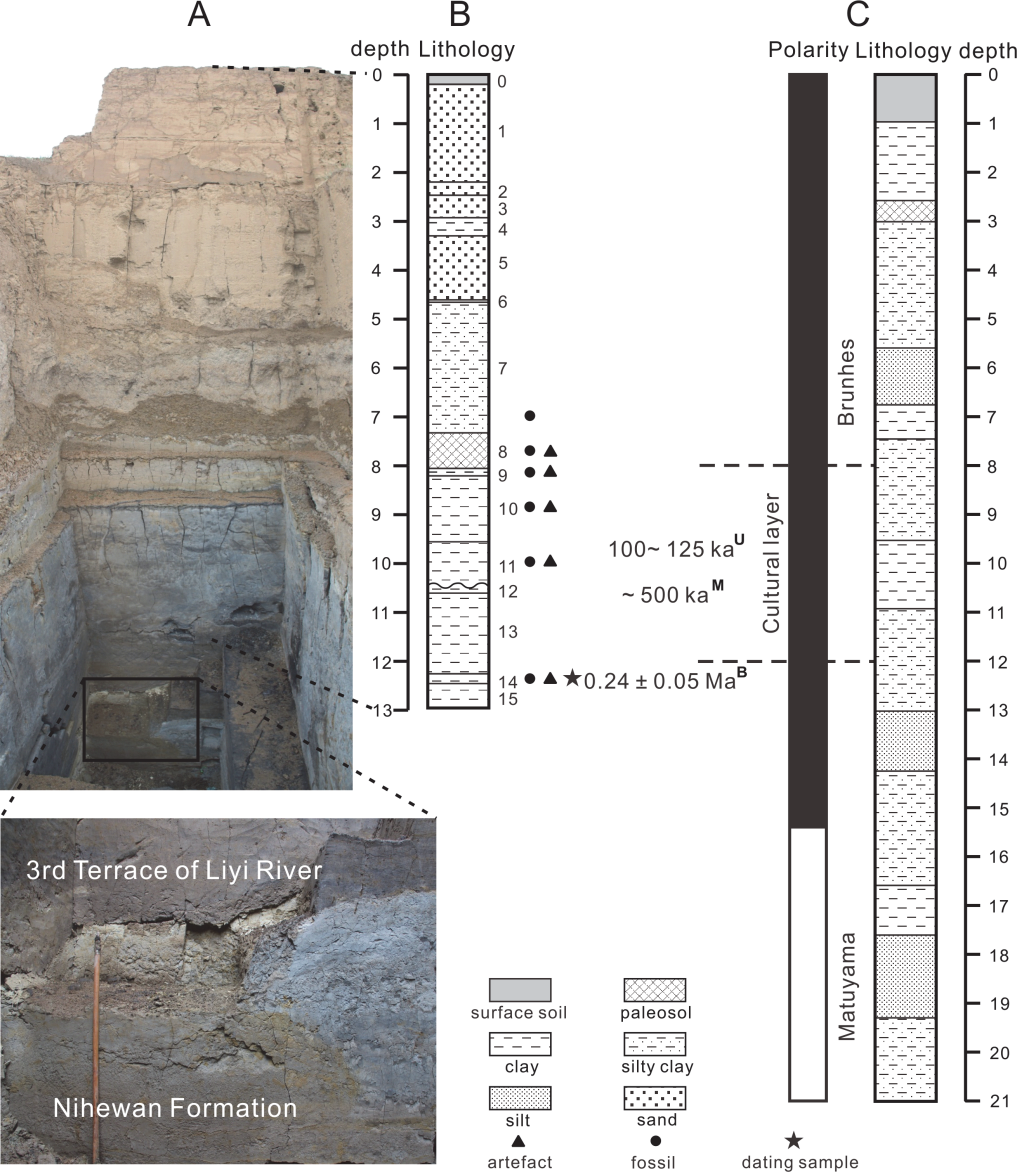
Stratigraphy of the Xujiayao-Houjiayao Paleolithic site. (A) Photograph of the cross-section revealed during the excavation in 2012, the lower left inset shows more clearly an unconformity believed to mark the boundary between the third terrace of Liyi River and the underlying Nihewan Formation. (B) Lithostratigraphic column of the cross-section exposed in 2007–2008 (∼1 m shallower than A) with the position of the dating samples and their ^26^Al/^10^Be burial dates. (C) Lithostratigraphic column and polarity of the cross-section from where samples for magnetostratigraphic studies were taken (modified from Løvlie et al. [15]), along with the magnetostratigraphic dates [17, 18]. Also given are the U-series dates on fossil teeth [7]. Superscripts on ages indicate the dating methods (B, Burial dating; U, U-series dating on fossils; M, Magnetostratigraphic dating).

However, further paleomagnetic studies by an international team of Sino-Norwegian scientists challenged the above consensus. Su et al. [14] and Løvlie et al. [15] collected samples with a reduced sampling interval and extended the study section to greater depth. The reversed polarity zone previously recognized by Liu et al. [12] was found to be much thicker (6–12 m), and thus re-assigned to the Matuyama Chron rather than to a brief excursion (Fig. 2C). As only one geomagnetic event was identified, Su et al. [14] first proposed that the cultural layer, being only 3 m above the B/M boundary, should be much older than previously estimated. Though Løvlie et al. [15] acknowledged the possibility that the cultural layer may be incorporated into younger fluvial sediments that overlie the much older fluvio-lacustrine Nihewan Formation, Fan et al. [16] later proposed a more specific age of early-middle Mid-Pleistocene for the site based on sedimentation rates deduced from other depositional sections in Nihewan Basin. By correlating the magnetic signature with the marine oxygen isotope records, Wang et al. [17, 18] assigned the top soil of the cross-section to S1 (ca. 129 ka) in the Chinese loess/paleosol sequence, and thus proposed a numerical age of ∼500 ka for the site, but suggested that the Paleolithic artifacts and mammalian fossils might be held within a much younger fluvial unit.

Scholars from the prehistoric community hold different viewpoints on the possibility of a much older X-H site. While Huang et al. [19] consider that the biostratigraphic correlations provide an age constraint too loose to negate the new paleomagnetic dating, Norton and Gao [20] prefer waiting for further evidence. On the other hand, Wu and Trinkaus [21] and Wu et al. [22] tend to insist on the biostratigraphic age estimate, Xing et al. [23] rely more on the U-series dating of fossils, and sharp criticisms of the paleomagnetic age as being too old to be realistic may be found in Xie [24], Wei [25], Wei and Wu [26, 27] and Wang et al. [28].

To address the decade-long controversy described above, and to date the cultural deposits directly, a cross-check by an independent and well-established radio-isotopic dating method is needed. Here we report the application of ^26^Al/^10^Be burial dating to the X-H site.

### Stratigraphy and samples

The X-H site (40°06′3″N, 113°58′41″E, 980 m above sea level) is situated on the west bank of the Liyi River, a tributary of the Sanggan River (Fig. 1). The Liyi River has cut the older Nihewan Formation and brought about new sediments, leaving a 200–500 m wide valley and 15–20 m high escarpments composed of three terraces. During three excavations in the 1970’s, sediments from an area of more than 1,500 m^2^ and to a depth of ∼12 m were removed. The hominin and mammalian fossils and lithic artifacts were recovered from a yellowish-green clay layer at depths of 8–12 m below the ground surface [1, 2].

In 2007 and 2008, researchers from Hebei Provincial Institute of Cultural Relics organized a new round of systematic excavation at the northwest corner of the site. The excavation pit was ∼10 m^2^ in area and ∼13 m in depth. The depositional sequence was divided into 15 layers (Fig. 2B). The upper Layers 1–6 are sterile and composed of interlayered fine sand, clay and silty clay. Layer 7 consists of silty clay with some mammalian fossils discovered in its lower horizon. Layer 8 is composed of a reddish brown paleosol, Layers 9–11 are bluish or yellowish brown clayey deposits, and the lower Layers 12–15 are greyish blue, dark grey or black sandy clay or clayey sand. Among them, Layers 8–11 and 14 are artifact- and fossil-bearing. The excavators defined two cultural units, the upper one (Layers 8–11, 7.2–10.3 m) should correlate to the cultural deposits of the previous excavations [1], the lower one (Layer 14, 12.1–12.3 m) is believed to be newly discovered, though no faunal and cultural difference between the two units can be identified [29, 30]. When carrying out field studies, for exploring the site’s extension the excavators sank a number of cores at the side and back of the cross-section with a Luoyang Spades (S1 Fig.). In this way it was found that the cultural deposits extend over an area of more than 1000 m^2^, and that the depositional sequence is more or less continuous. The continuity of the fossil and artifact-bearing layers is observable on the existing cross-section from the previous excavations (S2 Fig.), where they can be traced from one side to the other.

During a field investigation at the site, we found that Layer 14 contains sufficient coarse quartz grains for burial dating with ^26^Al and ^10^Be. A quartzose sand sample, HJY-QZ, was taken there. Among the lithic objects recovered from Layer 14, a quartzite gravel clast was selected and analyzed as HJY-ST.

For better illustrating the stratigraphic position of the two samples for dating, the three cross-sections given as Figs. 2, 3 and 4 in Jia and Wei [1] are presented here as S3 Fig., and their correlation with the new excavation is briefly discussed in the caption. Apart from the continuity of the cultural deposits as discussed above, the *in situ* formation of these deposits on the flood plain of Liyi River is supported by the fact that none of the stone artifacts (observed by the 4^th^ author of this paper) and only a few of the fossil bones [20] of the site bears any sign of fluvial wear. Taken together, we are assured that, in spite of the presence of gravelly or sandy lenses and depositional hiatuses as shown in S3 Fig., the above two samples are stratigraphically beneath the cultural deposits.

### Ethics statement

The sample HJY-ST was recovered during excavations carried out by one of the co-authors, F. Xie of Hebei Provincial Institute of Cultural Relics. We collected the quartzose sample, HJY-QZ, together with F. Xie and his colleagues. According to the current rules for protecting the archaeological sites, approval from the Provincial Institute of Cultural Relics is necessary, but no specific permission is required. The field work did not involve any endangered or protected species.

## Method and Experiments

The burial dating method is based on the buildup and decay of cosmogenic ^26^Al (t_1/2_ = 717 ± 17 ka) and ^10^Be (t_1/2_ = 1.39 ± 0.01 Ma) in the mineral quartz. These radionuclides are formed by secondary cosmic rays that penetrate into the ground surface and react with nuclei within mineral grains. The production rates of the two nuclides depend on several factors including altitude and geomagnetic latitude, but the ^26^Al/^10^Be ratio of about 6.75 remains basically constant [31]. As quartz mineral grains are gradually eroded from bedrock and transported through river networks, they are exposed to secondary cosmic rays for a sufficiently long time to acquire an inventory of ^26^Al and ^10^Be. If the quartz minerals are then rapidly and deeply buried in a river terrace or a cave, then the production of cosmogenic nuclides drastically slows down or even ceases, and each radionuclide decays according to its specific half-life. Because ^26^Al decays more rapidly than does ^10^Be, the ^26^Al/^10^Be ratio decreases exponentially over time with an apparent half-life of 1.48 Ma. This basic scheme offers a means of dating burial events of quartz minerals [32, 33].

The effective range of ^26^Al/^10^Be burial dating depends mainly on the precision of ^26^Al and ^10^Be measurements. With accelerator mass spectrometry (AMS) normally ^10^Be and ^26^Al can be measured at precisions of ∼1–3% and ∼4–7% (1σ, the same hereafter), respectively, leading to an uncertainty of ∼5–8% in ^26^Al/^10^Be and an absolute uncertainty of ∼70–120 ka in age results. The recent addition of a gas-filled magnet at PRIME Lab (Purdue University) has substantially improved the precision of ^26^Al measurements, making their uncertainties comparable to those for ^10^Be, and frequently leading to uncertainties in the ^26^Al/^10^Be ratio of <5%. With this level of precision, ^26^Al/^10^Be burial dating should be able to resolve the highly controversial issue concerning the chronology of the X-H site. If the site is really as old as suggested by the paleomagnetic studies, burial dating may provide supporting evidence for its validity. Otherwise, if the site is closer to the biostratigraphical age estimate, burial dating may give ages statistically indistinguishable from zero.

For HJY-QZ several kilograms of quartzose deposits were collected and rinsed with water to remove silt and clay. The remaining material was taken back to the laboratory for further treatment. HCl was added to dissolve the carbonate and phosphate components. For HJY-ST the gravel was crushed to submillimeter grains. For either sample type, grains of 0.2–0.9 mm were sieved out and then leached several times in 2% HF-2% HNO_3_ overnight with heating and agitation. Magnetic and gravimetric separations were performed to separate quartz from all other contaminating minerals. The quartz grains were further treated by repeated leaching overnight in 1% HF-1% HNO_3_ in an ultrasonic tank to ensure complete removal of meteoric ^10^Be.

Purified quartz samples were dissolved in 5:1 HF/HNO_3_, and spiked with ∼0.2 mg ^9^Be carrier. Aliquots were taken from the dissolved samples for Al concentration measurement by ICP-OES. After HF volatilization, Al and Be were separated and purified on ion-exchange columns in 0.4 M oxalic acid, precipitated as hydroxides, and transformed to oxides in a furnace at 1,100°C. BeO was mixed with niobium and Al_2_O_3_ with silver powder for ^10^Be/^9^Be and ^26^Al/^27^Al measurements by accelerator mass spectrometry (AMS) at PRIME Lab, Purdue University.

## Results

The ^26^Al and ^10^Be concentrations and calculated burial ages are presented in Table 1, and other experiment and measurement data related to the age calculation are given in S1 Table. The two samples yield consistent age results 0.26 ± 0.06 and 0.17 ± 0.12 Ma, respectively, with a weighted mean of 0.24 ± 0.05 Ma.

**Table 1:** Cosmogenic nuclide concentrations and burial ages for Xujiayao-Houjiayao site.

Sample	[^26^Al]_1_ (10^6^ at/g) †	[^26^Al]_2_ (10^6^ at/g) †	[^10^Be] (10^6^ at/g)	^26^Al/^10^Be	Burial age (Ma)
HJY-QZ	1.795 ± 0.097	1.712 ± 0.036	0.293 ± 0.005	5.879 ± 0.158	0.258 ± 0.055
HJY-ST	0.302 ± 0.030	0.320 ± 0.015	0.051 ± 0.002	6.202 ± 0.366	0.174 ± 0.119
Weighted mean					0.243 ± 0.050

† The [^26^Al]_1_ values were initially measured with relative counting errors of 5–10%. The [^26^Al]_2_ values were obtained by re-measuring the remaining Al_2_O_3_ powder after the installation of a gas-filled magnet into the AMS at PRIME Lab. The weighted mean of the two measurements, 1.722 ± 0.034 and 0.316 ± 0.013 (10^6^ at/g), respectively, were used to calculate the ^26^Al/^10^Be ratios and then the age results.

As in the case of other radioisotopic dating methods, the reliability of ^26^Al/^10^Be burial dating depends closely on a set of assumptions. The most important one is that samples be deposited with a ^26^Al/^10^Be ratio consistent with “simple steady-state erosion”. To meet this assumption, the quartz minerals must have experienced only one exposure-burial cycle in the past ∼10 Ma, otherwise the burial ages should be considered maxima. We cannot strictly exclude the possibility that the two X-H samples have experienced complicated exposure-burial histories, but the agreement between the two ages within measurement error and their relatively young ages render it unlikely.

The second assumption necessary for accurate burial dating is that the samples should be buried deeply enough to ignore post-burial production of cosmogenic nuclides by muons. Since the two samples of this study were taken from the same Layer 14 at a depth of 12.3–12.5 m, and their ages are quite young, the postburial production of cosmogenic nuclides can be securely neglected.

Because of the possibility for the samples to have experienced complicated exposure-burial histories, and also because the samples were collected from the lower cultural unit, their burial ages should be regarded as a maxima for the X-H site. Thus at 95% confidence, this site should be younger than 340 ka. On the other hand, the results presented here may indicate an age older than 140 ka for the site’s lower cultural deposits. However, due to uncertainties in the samples’ pre-burial histories, the younger age constraint is less stringent.

## Discussion

The weighted mean age of the two samples, 0.24 ± 0.05 Ma, is consistent with the recent post-IR IRSL dating of the site, which yielded a range of 161 ± 13 and 224 ± 22 ka for Layers 8–11 [34]. Considering the faunal and palynological evidence that is indicative of a period of cold climate [1, 20, 34], the results of this paper lend support to the proposition that the X-H site should correspond to Marine Isotope Stage (MIS) 6 (∼130–190 ka) [34]. Rather than the widely accepted previous age estimate of early Upper Pleistocene, this site is more likely late Middle Pleistocene in age.

Our radiometric age for the X-H site is significantly younger than the age of ∼500 ka suggested by paleomagnetic studies [14–18]. Though widely applied and highly useful in geochronological studies, it must be kept in mind that paleomagnetic stratigraphy is not an absolute dating method, and that extrapolation from even firmly dated magnetic boundaries relies on implicit assumptions of continuous and steady sedimentation that can be difficult to satisfy in fluvio-lacustrine sediments. Cited here as an example, a depositional hiatus marking a sedimentation break between 266 and 128 ka was recognized within the Haojiatai section at the eastern margin of Nihewan Basin [35]. At the X-H site, three erosional surfaces were recognized by Jia and Wei [1]. During the excavation of 2007–2008 and also during the latest one in 2012, two unconformities were identified, one between Layers 11 and 12 (See Fig. 2B), another one ∼0.7 m below the lower cultural unit. The latter one is believed to be the erosional unconformity between the third terrace of the Liyi River and the underlying Nihewan Formation (Fig. 2A) [24, 36, 37], which leads to the discrepancy between paleomagnetic stratigraphy and other dating methods. The results of this paper support strongly this interpretation.

The evolutionary position of Middle Pleistocene hominins in eastern Asia, generally referred to as archaic, earlier or pre-modern *H*. *sapiens*, is currently one of the hotly debated issues in paleoanthropology (e.g., [5, 38–41]). The presence of archaic *H*. *sapiens* in eastern Asia was traditionally considered to be limited to the past ∼200 ka, based mainly on biostratigraphic correlations and on U-series dating of fossils, the latter being known for its limited reliability. Over the past more than two decades, however, more reliable dating methods such as U-series dating of speleothem calcite has pushed back the dates of the key hominin and contemporaneous archaeological sites traditionally assigned to archaic *H*. *sapiens*, such as Tongzi [42], Maba [43], Miaohoushan [44], Chaoxian [45], and Localities 4 and 15 at Zhoukoudian [46, 47], more typically to 200–500 ka. The X-H hominin, dating to MIS 6, may thus be one of the youngest representatives of archaic *H*. *sapiens* in China. This hominin group was skilled at hunting horses [48], and their capacity of securing a regular source of animal fat and protein is strongly correlated with early evidence of modern human behavior [20]. Further studies of the site’s rich discoveries may provide more evidence for addressing the debated issue of Middle-Upper Pleistocene human evolution.

Since its first application for deriving river incision rates [49], ^26^Al/^10^Be burial dating has played an increasingly important role in studying long-term landscape evolution and early human evolution. This method is particularly promising in dating early hominin sites in China, where the lack of volcanic tuffs precludes the application of ^40^Ar/^39^Ar dating. By assigning a maximum age of 0.24 ± 0.05 Ma to a lower cultural horizon of the X-H site and thus controverting firmly the paleomagnetic dating, the results reported in this paper exemplify its strength. Along with the establishment of isochron technique to circumvent some of the geological complexities [50, 51], the recent improvement in the precision of ^26^Al marks an important methodological milestone. Burial dating with ^26^Al and ^10^Be is now a well-established and reasonably precise radio-isotopic dating method. Its further application will help to establish a reliable temporal framework for the mode of human evolution in East Asia.

## Supporting Information

S1 Fig A photo showing a survey for boundaries of the X-H site at the side and back of the new excavation using a Luoyang Spade (a kind of portable coring tool). (JPG)

S2 Fig Photograph showing the extant cross-section from the excavations in the 1970’s (left) and the recent one (right). (TIF)

S3 Fig Three cross-sections in Jia and Wei [1]. A, B and C correspond to the Figs. 2, 3, and 4, depicting a depositional sequence excavated in 1974, one ∼10 m to the south, and another one ∼40 m further south, respectively. By synthesizing the three cross-sections, the authors proposed a stratigraphical sequence as follows: **U8**. Modern fluvial deposits or brown sandy topsoil (ceramic fragments bearing), 0.3–0.5 m thick, with a gradual transition to the underlying layer (not given in the figures.). **U7**. Sandy or yellowish brown loess, with occasional sand sub-layers, with horizontal laminae, 3–5 m thick (see Layers 3, 9 and 11 in S2A, S2B and S2C Fig., respectively). **U6**. Yellowish brown silt (reddish), without clear horizontal but with vertical laminae, with a few small gravels and gravelly sandy lens, bearing *Coelodonta antiquitatis* tooth fragments, *myospalax fontanieri* fossils and Ostrich eggshells, up to 5 m thick (Layer 8 in S2B and S2C Fig.). **U5**. Reddish silt, with a few gravels, 1–3 m thick (Layer 7 in S2C Fig.). **U4**. Yellowish green sandy clay, 4 m thick, with a sublayer of 0.1 m-thick sandy concretion at the top (Layer 6 in S2C Fig.). **U3**. Brownish red clay (paleosol), 0.3 m thick (Layer 5 in S2C Fig.). **U2**. Yellowish green (grayish brown in the north) sandy clay, with a few gravels, with sandy nodules at the top, ∼6 m thick. All the stone artifacts and most of the fossils were found in this layer (Layers 2, 7 and 4 in S2A, S2B and S2C Fig., respectively). **U1**. Grayish blue, grayish green or grayish brown clay, horizontally stratified, with some localized thin layers of grayish white clay or grayish yellow silt. The exposed thickness is 4–8 m (Layers 1, 1–6 and 1–3 in S2A, S2B and S2C Fig., respectively). From the above description, we may find that the cultural deposits (U2), a layer of paleosol (U3) and the lowest depositional unit (U1) of the excavations in 1970’s correlate fairly well with Layers 9–11, Layer 8 and dark colored Layers 12–15, respectively, of the new excavation as described in the text.
(TIF)

S1 Table Experiment and measurement data for Xujiayao-Houjiayao site. (DOCX)
