# Supplementary figures and images for: 26Al/10Be Burial Dating of Xujiayao-Houjiayao Site in Nihewan Basin, Northern China
Source: PLoS One. 2015 Feb 23;10(2):e0118315. doi: 10.1371/journal.pone.0118315 (PMC4338100; doi:10.1371/journal.pone.0118315)

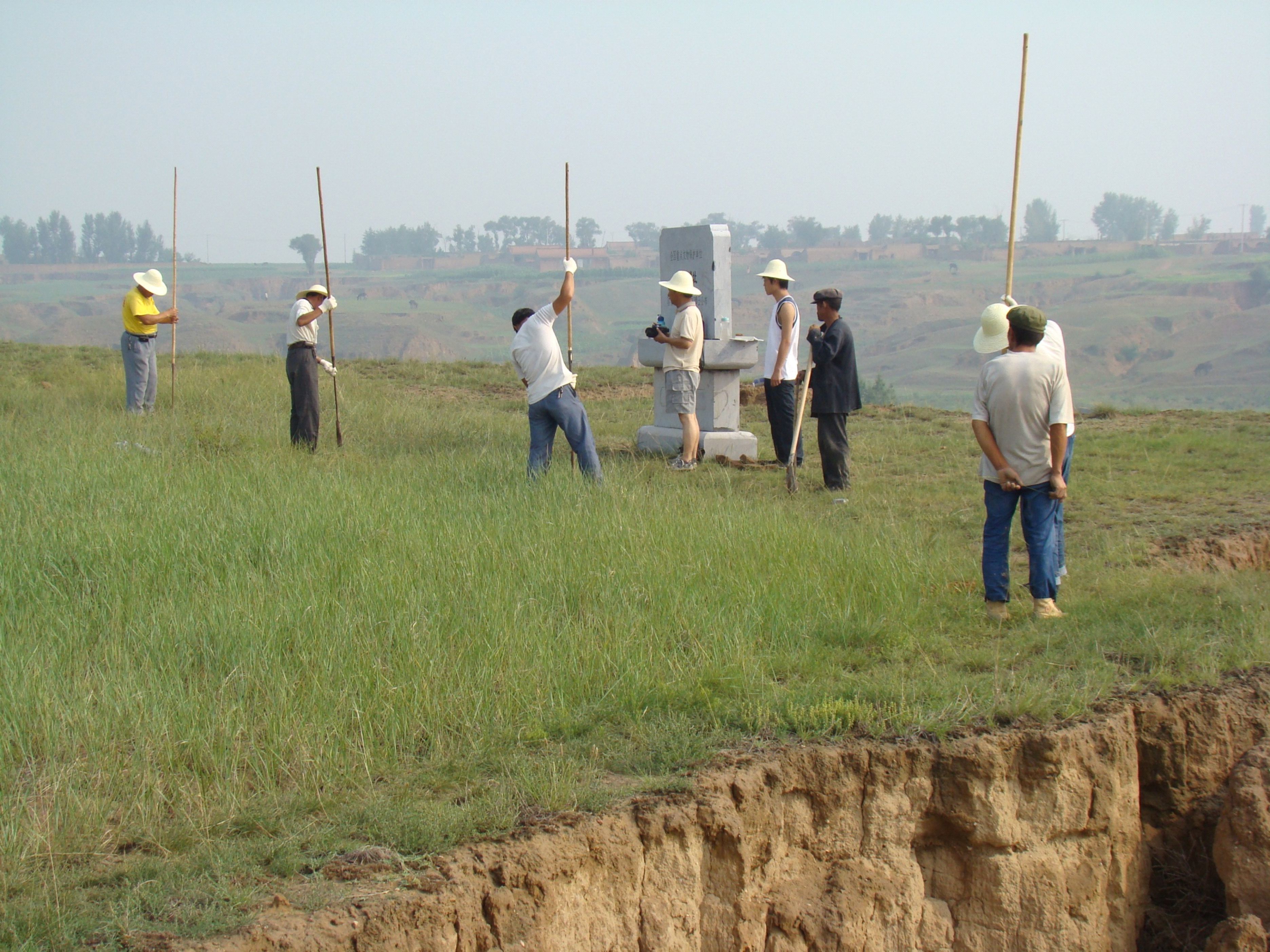

Supplement: S1 Fig — (JPG) [file pone.0118315.s001.jpg]

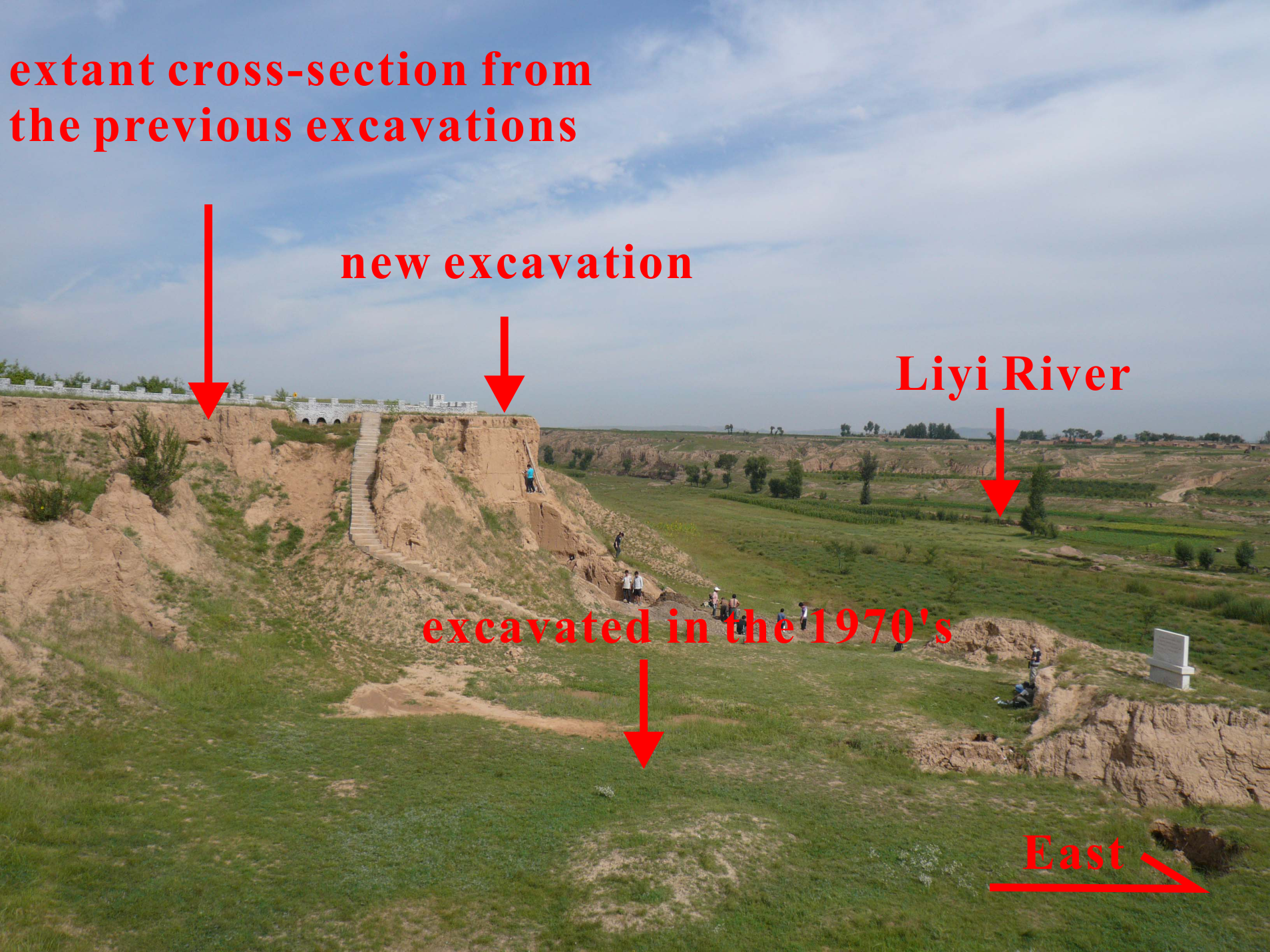

Supplement: S2 Fig — (TIF) [file pone.0118315.s002.tif]

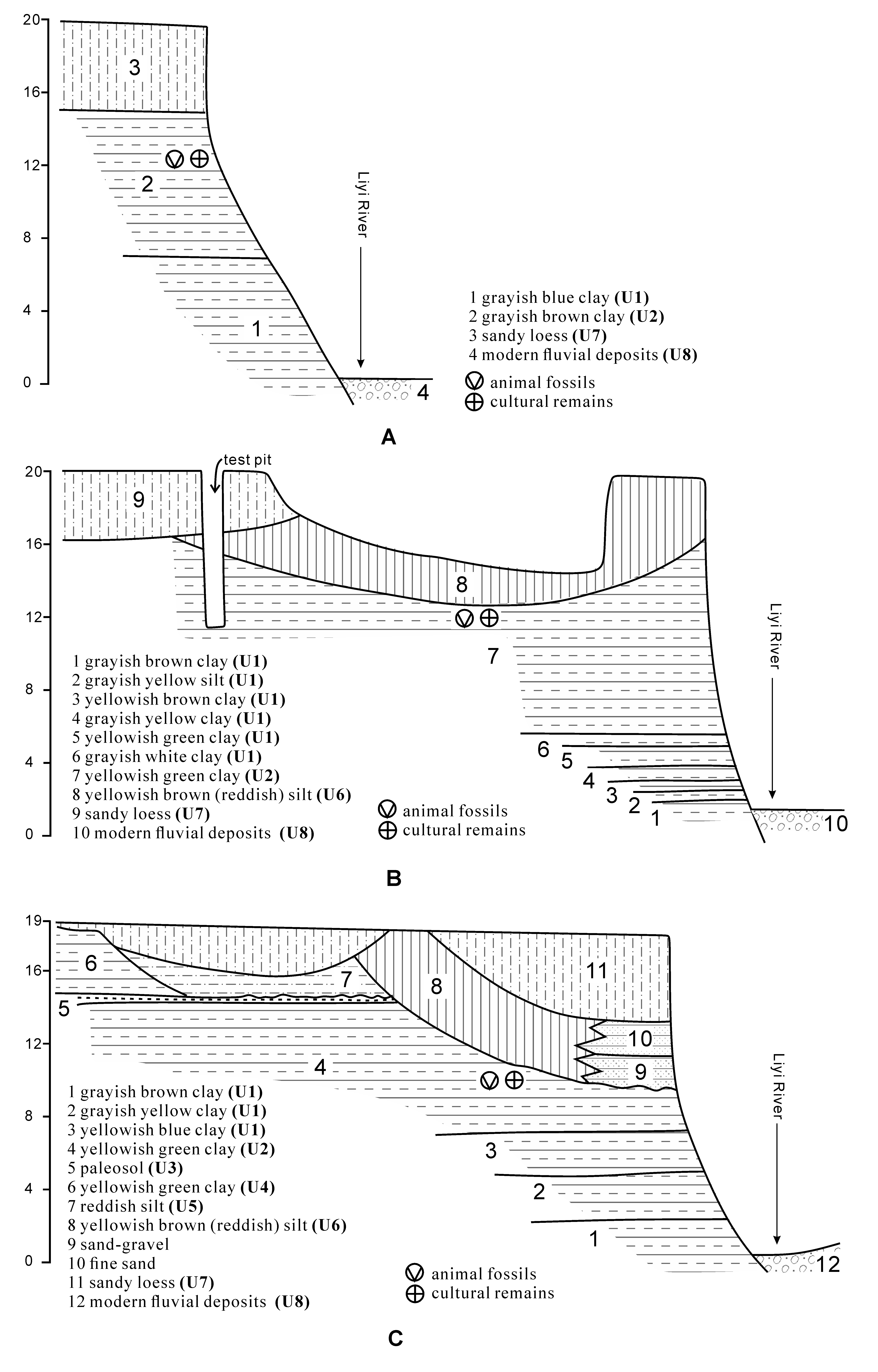

Supplement: S3 Fig — A, B and C correspond to the Figs. 2, 3, and 4, depicting a depositional sequence excavated in 1974, one ∼10 m to the south, and another one ∼40 m further south, respectively. By synthesizing the three cross-sections, the authors proposed a stratigraphical sequence as follows: U8. Modern fluvial deposits or brown sandy topsoil (ceramic fragments bearing), 0.3–0.5 m thick, with a gradual transition to the underlying layer (not given in the figures.). U7. Sandy or yellowish brown loess, with occasional sand sub-layers, with horizontal laminae, 3–5 m thick (see Layers 3, 9 and 11 in S2A, S2B and S2C Fig., respectively). U6. Yellowish brown silt (reddish), without clear horizontal but with vertical laminae, with a few small gravels and gravelly sandy lens, bearing Coelodonta antiquitatis tooth fragments, myospalax fontanieri fossils and Ostrich eggshells, up to 5 m thick (Layer 8 in S2B and S2C Fig.). U5. Reddish silt, with a few gravels, 1–3 m thick (Layer 7 in S2C Fig.). U4. Yellowish green sandy clay, 4 m thick, with a sublayer of 0.1 m-thick sandy concretion at the top (Layer 6 in S2C Fig.). U3. Brownish red clay (paleosol), 0.3 m thick (Layer 5 in S2C Fig.). U2. Yellowish green (grayish brown in the north) sandy clay, with a few gravels, with sandy nodules at the top, ∼6 m thick. All the stone artifacts and most of the fossils were found in this layer (Layers 2, 7 and 4 in S2A, S2B and S2C Fig., respectively). U1. Grayish blue, grayish green or grayish brown clay, horizontally stratified, with some localized thin layers of grayish white clay or grayish yellow silt. The exposed thickness is 4–8 m (Layers 1, 1–6 and 1–3 in S2A, S2B and S2C Fig., respectively). From the above description, we may find that the cultural deposits (U2), a layer of paleosol (U3) and the lowest depositional unit (U1) of the excavations in 1970’s correlate fairly well with Layers 9–11, Layer 8 and dark colored Layers 12–15, respectively, of the new excavation as described in the text. (TIF) [file pone.0118315.s003.tif]
