# Supplementary material for: 26Al/10Be Burial Dating of Xujiayao-Houjiayao Site in Nihewan Basin, Northern China
Source: PLoS One. 2015 Feb 23;10(2):e0118315. doi: 10.1371/journal.pone.0118315 (PMC4338100; doi:10.1371/journal.pone.0118315)
Supplement: S1 Table — (DOCX) [file pone.0118315.s004.docx]

**Table S1.** Experiment and measurement data for Xujiayao-Houjiayao site

| Sample | Sample size (g) | Be spike (g) * | ^10^Be/^9^Be (×10^-15^) | ^27^Al  (µg g^-1^ quartz) | [^26^Al/^27^Al]_1_ (×10^-15^) † | [^26^Al/^27^Al]_2_ (×10^-15^) † |
| --- | --- | --- | --- | --- | --- | --- |
| HJY-QZ | 49.7 | 0.267 | 771 ± 14 | 72.50 | 1110 ± 60 | 1065 ± 23 |
| HJY-ST | 43.9 | 0.267 | 127 ± 5 | 90.84 | 149 ± 15 | 162 ± 7 |

* Be spike concentration is 1069 ± 8 ppm

† The [^26^Al/^27^Al]_1_ values were from the initially measurements. The [^26^Al/^27^Al]_2_ values were obtained by re-measuring the remaining Al_2_O_3_ powder with the installation of a gas-filled-magnet into the AMS. ^10^Be/^9^Be ratios in blanks was 10 ± 2 ×10^-15^ and ^26^Al/^27^Al blank ratio was 4 ± 4 ×10^-15^ for the initial measurement and 3 ± 1 ×10^-15^ for the re-measurement.
